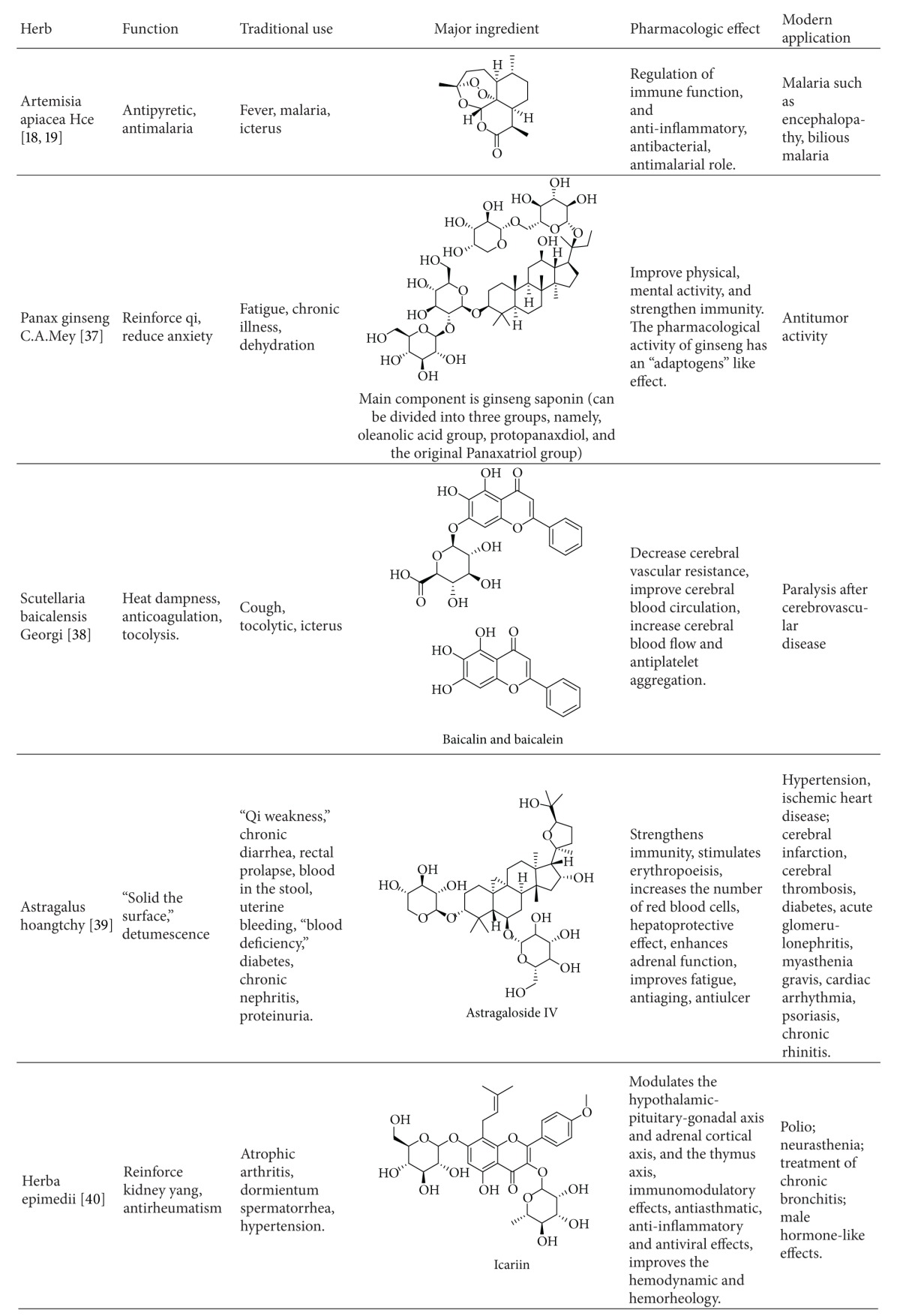# The Relationship between Traditional Chinese Medicine and Modern Medicine

**DOI:** 10.1155/2013/153148

**Published:** 2013-07-30

**Authors:** Jingcheng Dong

**Affiliations:** ^1^Institute of Integrated Medicine, Huashan Hospital, Fudan University, Shanghai 200040, China; ^2^Department of Integrated Medicine, Huashan Hospital, Fudan University, Shanghai 200040, China

## Abstract

The essence of the traditional Chinese medicine has always been the most advanced and experienced therapeutic approach in the world. It has knowledge that can impact the direction of future modern medical development; still, it is easy to find simple knowledge with mark of times and special cultures. The basic structure of traditional Chinese medicine is composed of three parts: one consistent with modern medicine, one involuntarily beyond modern medicine, and one that needs to be further evaluated. The part that is consistent with modern medicine includes consensus on several theories and concepts of traditional Chinese medicine, and usage of several treatments and prescriptions of traditional Chinese medicine including commonly used Chinese herbs. The part that is involuntarily beyond modern medicine contains several advanced theories and important concepts of traditional Chinese medicine, relatively advanced treatments, formula and modern prescriptions, leading herbs, acupuncture treatment and acupuncture anesthesia of traditional Chinese medicine that affect modern medicine and incorporates massage treatment that has been gradually acknowledged by modern therapy. The part that needs to be further evaluated consists not only the knowledge of pulse diagnosis, prescription, and herbs, but also many other aspects of traditional Chinese medicine.

## 1. Introduction

The essence of traditional Chinese medicine has always been the most advanced and experienced medicine in the world. Its vast system is full of practical medical technology and proven experiences that have been gradually incorporated into modern medicine; it has knowledge that can impact the direction of future medical development, yet its knowledge is simple enough to fit changing times and varied cultures. The basic structure of traditional Chinese medicine is composed of the following three parts: the part that is consistent with modern medicine, the part that is involuntarily beyond modern medicine, and the part that needs to be further evaluated or abandoned.

## 2. The Part That Is Consistent with Modern Medicine

In the 16th century, what is now known as “western medicine” was introduced to China but was not commonly used and thus had little effect. It was not until the Opium War that “western medicine” began to develop in China. Therefore, prior to that event, traditional Chinese medicine was always the leading force of medical care in China. 

The key to successful medicine lies in its efficacy. A popular Chinese saying states “Excellence is from experience.” The foundation of traditional Chinese medicine is based on 5,000 years of practice and experiences. With the founding of a new China in 1949, western medicine in the region also began to play a large role in medical care. As modern (westernized) medicine's impact increased, it led to the development of “integrative medicine” at the end of the 1950s. Theories, therapeutic principles, technologies, and understanding of the life sciences were elaborated, and the basic structure of traditional Chinese medicine also became clearer. Most importantly, traditional Chinese medicine began to reach a common point with modern medicine.

### 2.1. Consensus on Several Theories and Conceptions of Traditional Chinese Medicine

For example, “the essence of kidney” is a core concept of traditional Chinese medical theory. Studies by Shen showed that “kidney deficiency syndrome” is equivalent to the aging in modern medicine, regardless of its external performance and internal changes [1–3]. Studies by Chen et al. showed that what is known as “heart qi deficiency” is associated with cardiac insufficiency in modern medicine [4–6]. 

### 2.2. Consensus on Inspection, Auscultation-Olfaction, Inquiry, and Palpation Method

There are four main components of traditional Chinese medicine diagnosis [7, 8]. Inspection is a method to determine the medical status by visually examining the changes in appearance and the movement of the whole body or part of the body. Generally speaking, inspection includes examination of the tongue as well as the observation of a patient's external appearance, in order to recognize the internal and external manifestations of disease. Visual examination is consistent with modern medicine's emphasis on observation of a patient appearance and movement. For example, a pale lip can indicate anemia in modern medicine. 

The use of auscultation-olfaction, including listening to the sounds and smelling for odors, is also consistent with modern medicine. If a patient speaks loudly and powerfully, the physician can determine that the patient is full of energy. Conversely, if a patient's voice is weak or he does not want to talk, it is most likely that the patient's diseases are caused by “deficiency.” A patient's bodily odors can also reveal much about the respective illness. If a patient has a rotten apple like odor, it is likely diabetic ketoacidosis. Inquiry refers to the gathering of a thorough history and reviewing the systems. These include physical and mental feelings, life history, family history, past medical history, onset time, and the present symptoms, which are widely used both in traditional medicine and modern medicine. 

### 2.3. Consensus on Several Treatments of Traditional Chinese Medicine

Therapeutic studies show that Chinese medicine and modern medicine have reached a consensus on the methods of diaphoresis, purging, vomiting, warming, clearing, neutralizing, eliminating, and reinforcing (eight commonly used treatments in traditional Chinese medicine). For instance, the “reinforcing method” is able to improve neuroendocrine regulatory function, enhance the body's ability to combat stress, adjust immune function, and improve the body's systemic function. Diaphoresis is able to promote perspiration and defervescence as well as play a role in reducing inflammation among other methods adopted by modern medicine. 

### 2.4. Consensus on Several Prescriptions of Traditional Chinese Medicine and Commonly Used Chinese Herbs

Illnesses cured by many ancient Chinese compounds are similar to certain diseases or conditions also seen in modern medicine and are summarized in Table 1. 

There are many Chinese herbs either given as single drugs or as formulations that have been effective treatments for particular illnesses for thousands of years. With the development of modern medicine, the pharmacology and mechanism of action of many of these Chinese herbs have been determined, so that traditional Chinese medicine has gradually formed a consensus with modern medicine (Table 2). 

## 3. The Part Ahead of Modern Medicine Involuntarily

Traditional Chinese medicine is a practical medicine built on experience. The theoretical system is built by means of an ancient plain materialist philosophy, the method of “syndrome differentiation,” and the use of natural means to treat illness rather than emphasizing consistency with contemporary science and technology [9, 10]. Unlike modern medicine that has view that “Nature that cannot be certified cannot be conquered,” keeping abreast of requirements of contemporary science, traditional Chinese medicine with the overall old and simple features involuntarily gives awareness beyond the era. The so-called “integrative medicine” is able to reveal this essence continually. 

### 3.1. Several Advanced Theories and Important Conceptions of Traditional Chinese Medicine

In terms of medical theories and ways of thinking, traditional Chinese medicine has a significant contribution to modern medicine. For example, the theory of the “correspondence between man and universe” in traditional Chinese medicine is the unified outlook of body and environment. What are known as the “biological life” theory and the “biological clock” theory in modern medicine refers to the patterns of hormone secretion, and treating diseases according to the place of origin are all the embodiment of “correspondence between man and universe.” The views of “unity of opposites” and “balance amongst dynamic forces” indicate the existence of universal laws in human life. Although these views are emphasized in modern life science, both approaches differ from traditional Chinese medicine because traditional Chinese medicine always considers these views as its guiding ideology and as fundamental law. Therefore, the cognitive degrees of Chinese medicine and western medicine in these views are of difference. 

For example, studies by Shen show that “kidney deficiency” is due to neuroendocrine disturbance and preaging of the function of the hypothalamic-pituitary axis, which is consistent with the hypothesis that “the senile bell lies in hypothalamus” proposed by Everiff in the 1980s [11]. However, the practice of traditional Chinese medicine precedes modern medicine by thousands of years. Our study found that the knowledge of “chronic disease involving kidney” in traditional Chinese medicine has a profound connotation in modern life sciences. Many diseases, such as airway inflammatory diseases, with long-term recurrence, often exacerbate the pathological changes in the anti-inflammatory system and HPA axis. Patients suffering from these are known to have significant “kidney deficiency.” We summarized many theories and concepts with advanced knowledge in traditional Chinese medicine in Table 3. 

### 3.2. Several Relatively Advanced Treatments of Traditional Chinese Medicine

“Same treatment for different diseases,” one of the most important and characteristic concepts in traditional Chinese medicine reflects the spirit of “syndrome differentiation and treatment” and refers to the thought that different diseases at certain stages of development show the same pathogenesis. And therefore, the same therapeutic principles can be applied to diseases. For example, according to modern medical diagnosis, different diseases are able to be cured by “reinforcing kidney and replenishing qi” as long as they are attributed to “kidney-qi deficiency” syndrome. Modern research has shown that this concept of “same treatment for different diseases” is likely to be one of the directions of modern medicine in the future. Because some of the “walls” that exist between the diseases are not always reasonable, the reason for division is often based on anatomy, rather than etiological and pathological changes. Studies have shown that different diseases with similar syndrome often have common pathological and physiological changes. It is easier to achieve better effects by using the same prescription because different diseases may have comparable changes at the cellular, molecular, and genetic levels, and drug targets or the target groups may be similar across various diseases. 

### 3.3. Several Relatively Advanced Formula and Modern Prescriptions

#### 3.3.1. Realgar-Indigo Naturalis Formula

**Figure 1:**
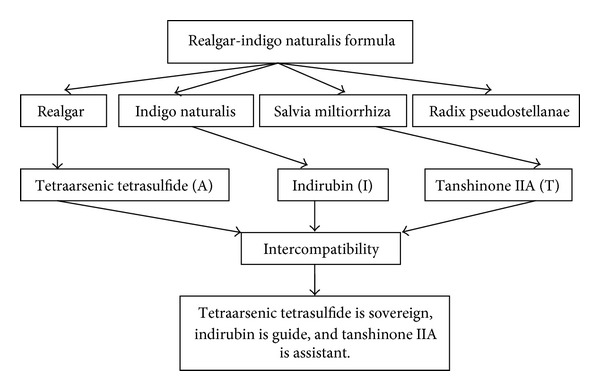
Realgar-Indigo naturalis formula.

Acute promyelocytic leukemia (APL) has a poor prognosis. In the 1980s, a famous Chinese medical expert in China, Doctor Shi-lin Huang, designed a Realgar-Indigo naturalis formula (RIF), in which, realgar, a mined ore, is the principal element and Indigo naturalis, Salvia miltiorrhiza, and Radix psudostellariae are adjuvant components to assist the effects of realgar. The main components of RIF are realgar, Indigo naturalis, and Salvia miltiorrhiza, with tetraarsenic tetrasulfide (A), indirubin (I), and tanshinone IIA (T) as major active ingredients, respectively [12–14]. Multicenter clinical trials showed that a complete remission rate of 96.7% [15] to 98% [16] and a 5-year overall survival rate of 86.88% [17] were achieved in APL patients receiving RIF, with moderate adverse effects such as gastrointestinal discomfort and rash. Doctor Zhu Chen clarified the molecular mechanism of the compound Huangdai tablets in the treatment of APL in 2008 from the perspective of molecular biology and biochemistry in the renowned journal Proceedings of the National Academy of Sciences of the United States of America (PNAS). The results show that As4S4 induces degradation of the cancer protein, thus reversing the increase in cancer cells and making them differentiate and mature. Tanshinone and indirubin promote the ubiquitination of the oncoprotein and accelerate its degradation, further promoting the differentiation and maturation of the leukemia cells and inhibiting cell cycle and proliferation of cancer cells. Animal studies also showed that the use of natural indigo after realgar substantially reduced toxicity. These reflect the concepts of typical “minister” (the place inferior to “monarch” drug) and “assistant” (the place inferior to “minister” drug) effect. Tanshinone and indirubin increase the production of channel proteins that deliver As4S4, which significantly increase the concentration of As4S4 in leukemia cells, improving its efficacy. Both tanshinone and indirubin play the role of “guide” (the place inferior to “assistant” drug). The compound Huangdai has a synergistic effect with the other components greater than its three individual components by the joint application of each component (Figure 1). 

#### 3.3.2. Airway Stabilizer Solution

The airway stabilizer solution created by Doctor Jing-cheng Dong is based on Xiao Qing Long Tang (Little Dragon Decoction) and Ding Chuan Tang (Anti-asthmatic Decoction), composed of ligusticum wallichii, ginkgo biloba, skullcap, Asarum, ginger, almonds, earthworm, rehmannia, and magnolia. It is effective in the clinical treatment of bronchial asthma and variability of cough. The various components and the basis for its efficacy are rooted in traditional Chinese medical theory, and the composition of the drug may be the main material basis for its efficacy (Table 4). 

### 3.4. Several Leading Herbs

The study of Chinese medicine is driven in part by the development of modern pharmacology. For instance, Artemisia annua was recorded in the “*Handbook of Prescriptions for Emergency*” (written by Hong Ge in Eastern Jin Dynasty) as a drug used to treat malaria. Modern research has shown that it has an antimalarial active ingredient (the peroxide group of sesquiterpene lactone) consisting of only 3 elements, carbon, hydrogen, and oxygen. It is a completely different novel compound compared to known antimalarial drug structures. This finding overturned the judgment of experts who insisted that the structure of the antimalarial drugs must have a nitrogen-containing heterocyclic ring [18, 19]. It helps provide a direction for the design and synthesis of new drugs. In addition, ginseng, skullcap, astragalus, epimedium, and other herbs were also each shown to have a unique effect (Table 5). 

### 3.5. Acupuncture Treatment and Acupuncture Anesthesia That Affect Modern Medicine

Those who practice acupuncture approach the treatment by looking at pathology in the whole body. According to different body conditions, varied acupoints and manipulations are selected. The selection influences multiple targets and many diseases and stimulates the body to treat diseases, affecting the pathological process and improving physique. 

This adjustment is accomplished by the integration of the central nervous system, including cortex recombination, neural plasticity, and release of various neurotransmitters and hormones [20, 21]. The basis of acupuncture may be in changes in gene expression. 

Acupuncture anesthesia is a method used to prevent surgical pain and relieve physiological dysfunction. It is suitable for those who are allergic to narcotic drugs. Since 1958, Shanghai No. 1 People's Hospital used it instead of narcotic drugs to perform tonsillectomies. Since then, acupuncture anesthesia has been passed from general usage to selected application. According to its clinical effect and scientific evaluation, acupuncture anesthesia is effectively used in thyroid surgery, surgery of posterior cranial fossa, craniocerebral operation, anterior cervical surgery, pulmonary resection, caesarean section, tubal ligation, and tooth extraction. It is also used in some surgeries with uncertain results like hysterectomy, caldwell-luc operation, subtotal gastrectomy, and strabismus surgery. It has not proved to provide effective anesthesia in limb surgery and perineal surgery. 

The basis of acupuncture anesthesia is the adjustment to inhibit large pain pathways by the negative reflection of spinal pain [22–24]. The acupuncture signal and the pain signal from the pain region transmit impulses into the brain. Acupuncture stimulates the antipain material to reduce the pain. Endogenous opioids participate in this process due to the increased release of opioids. Several neurotransmitters are related to acupuncture anesthesia, and some relatively central cerebral nuclei were found. CCK-8 has the negative-reflection-to-opioid effect, which is the vital factor of acupuncture and morphine. The effect of electric acupuncture depends on the balance of central opioid and CCK-8. The acupuncture signals can be reflected on some regional area of the brain to deal with the injury stimulations, which might be the physical basis of acupuncture anesthesia. Different frequencies of electric acupuncture differentiate the pain-relieving effects, which may be related to the specific expression of central genes. Two Hz electric acupuncture is widely used in the brain treatment, while 100 Hz has a narrow extension in the brain treatment. Psychological factors are not the deciding factors for success, but they are also quite important. 

### 3.6. Massage Treatment That Is Gradually Acknowledged by Modern Medicines

Massage treatment utilizes the particular skills with hands or limbs to practice manipulation on the surface of the body. Therefore, it has the direct effect of activating blood and dispersing stagnation, smoothing tendons, and improving malformation. On the other hand, massage reflectively influences the neuro and body fluid by acupoint-meridian-viscera network. Clinical researches show that appropriate manipulations can result in improvement of outcomes [25]. 

Modern research studies show that massage can promote blood and lymph circulation, increase metabolism, and assist the repair of soft tissue injury [26]. The diastolic function of the heart improves, and arteriole function improves as well after massage. The total cell count increases, while lung function improves. The content of catecholamines in plasma decreases so that the autonomic nervous system is inhibited, which can cause a reduction in pain [27]. Massage can effectively increase digestion in the stomach, adjust the secretion of stomach fluid, and release the spasm of smooth muscle [28]. When ST36 and BL23 are manipulated, the function of periphery blood T lymphocyte elevates. Rubbing manipulation, embrocation, and kneading manipulation could increase the elasticity and glossiness of skin. 

In short, skilled massage manipulation can effectively prevent diseases by the combination of static strength and motive force in a localized point or throughout the entire body. 

## 4. Some Parts That Need to Be Newly Recognized or Abandoned

In the system of human science, including medical science, concepts change from new to old, and ideas become theories and facts. All the concepts and rules indicate the stability. However, this kind of stability is conditional, partial, and relative, while instability is absolute and unconditional. Any scientific systems should confess their demerits and rectify them so as to generate new concepts, methods, and theory. Meanwhile, the initial phase of modern medicine and traditional Chinese medicine were founded in specific cultures and eras. So TCM theory should be divided into two parts, one of which needs to be promoted, the other of which need to be abandoned. Integrative medicine is an important way to achieve this [29]. 

### 4.1. About Palpation

“Cunkou pulse” should be newly recognized. It belongs to the “Lung Meridian of Hand-Taiyin.” “*Classic of Questioning*” (written by Qui Bian in the Warring StatesPeriod) put forward that “Cunkou pulse” can detect diseases of the whole body. “Lung” controls whole meridians, is the master of “qi”, and is the origin of “qi” and “blood.” Therefore, it can reflect all the changes of viscera, meridians and “qi-blood” to diagnose diseases. “*The Internal Canon of Medicine*” (written in Huang in the Warring States period) pointed out that meridians include meridians and collaterals, not vessels. The pulse we palpate originates from the heart, which beats. The meridian cannot beat, so it should not be able to be palpated. The pulse is only one kind of syndrome. Not all diseases can be presented by the pulse. Therefore, palpation is part of signs, but one should be careful when palpation is the only examination method. 

### 4.2. About Formula Prescription

Although formulary prescriptions are beneficial in medicine, they still have some demerits. It is hard to distinguish the “assistant” and the “guide” in some prescriptions, and therefore, it is necessary to conduct further research. “*Compendium of Materia Medica*” (written by Si-miao Sun in the Ming Dynasty) has lots of prescription to treat sterility. If a sterile female drank a cup of rain water in the spring, she would become fertile. If a sterile female stole a lamp from a rich person's bed, she would also become fertile. Even broom, dishwater, ashes on widow's bed, the wood in toilet, the trees fired by thunder, and the rope hung by itself were thought to treat diseases. 

### 4.3. About Chinese Medicine

Si-miao Sun (a well-known doctor in Chinese history) recorded saltpeter as bitter and hot in taste, extremely cold, and nontoxic. Modern researchers find saltpeter contains nitrocompound which may cause liver cancer. Also Pollia was recorded as bitter and hot in taste, warm and nontoxic, although it contains aristolochic acid which may cause kidney failure, lymphoma, kidney cancer, and liver cancer.

## 5. Conclusion

China is a nation composed of many ethnic groups, many with their own subcultures, beliefs, and history. Because of this, it is important to note that traditional Chinese medicine should have two concepts: firstly, it only refers to traditional medicine in the Han nationality; secondly, it is the sum of traditional medicines of all nationalities in the Chinese mainland. With respect to the structure and characteristic of traditional medicines, they can be divided into three parts: the knowledge and facts in agreement with modern medicine, the knowledge and practices not recognized in modern medicine that may be valuable in the future practice of modern medicine, and finally, the component of traditional medicine that has been adequately disproven and should be abandoned from future medical practice.

## Figures and Tables

**Table 1:** Several ancient herbal formulas that form consensus with modern medicine.

Formula	Origin	Composition	Function	Traditional application	Modern application
Ginseng Decoction [30]	Shiyao Shenshu from Xiuyuerubanjing Houlu	Large ginseng 20–30 g	Invigorating qi to prevent prostration	It is seen after hemorrhage, ulcer, and sore. Those who get qi and blood deficiency, pale complexion, aversion to cold with fever, cold limbs, spontaneous sweating or cold sweating, and faint pulse catch this kind of disease.	Hemorrhagic or cardiogenic shock

Sini Decoction [16, 31]	Taiyang meridian syndrome from Treatise on Febrile Diseases	Radix Glycyrrhizae Preparate (monarch, two liang), Rhizoma Zingiberis (minister, one and half liang), Radix Aconiti Preparata (assistant, one piece, uncooked)	Warming the middle energizer to dispel cold, Rescuing from collapse by restoring Yang	Shanghan Taiyang disease which is mistakenly treated by sweating method damages yang, yangming, taiyin, shaoyin, jueyin and cholera, displaying such symptom as deadly cold hand and foot, aversion to cold, vomiting and no eagerness to drink, hypogastralgia and diarrhea, spiritual deficiency and always sleeping, white and slippery tongue coating, faint pulse, and such diseases as plague, malaria, Jue disease, Collapse disease and pain disease. All above belong to yin syndrome.	Multiple shocks

Zhenwu Decoction [15]	Shaoyin meridian syndrome from Treatise on Febrile Diseases	Poria cocos, Paeonia Lactiflora Pall, Ginger (sliced) each three liang, Atractylodes macrocephala koidz (two liang), Radix Aconiti Lateralis Preparata (one piece, processed)	Warming yang to promote diuresis	It directs to deficiency of spleen-yang and kidney-yang and Water and Dampness Retention. The syndromes show as follows: dysuria; heavy limbs with pain; hypogastralgia and diarrhea; limb swelling; white tongue coating and no eagerness to drink; deep pulse; taiyang-diseases which are overused sweating method; edema due to yang insufficiency; sweating but not relieve the symptom; fever; epigastric throb; dizziness; trembling body.	Cardiogenic or renal edema

Yupingfeng Powder [17]	Spontaneous Sweating Decoction from Danxi's experiential therapy	Saposhnikovia divaricata, Radix astragali, one liang each, Atractylodes macrocephala koidz (two liang) Three qian. The above with one and half cup of water and three pieces of ginger	Tonifying Spleen and supplementing defending qi; Consolidating exterior for arresting sweating	It is called by TCM immunomodulator. It treats spontaneous sweating due to deficiency of yang, susceptibility of pathogenic wind; body injury by wind, rain, cold and dampness, and withered skin; sweating and disgusting wind; pale complexion, pale tongue, and thin-white coating, floating and deficient pulse. It also treats deficient people with loosened striae and susceptibility of pathogenic wind.	Rising the immune function

**Table 2:** Consensus of several commonly used herbs.

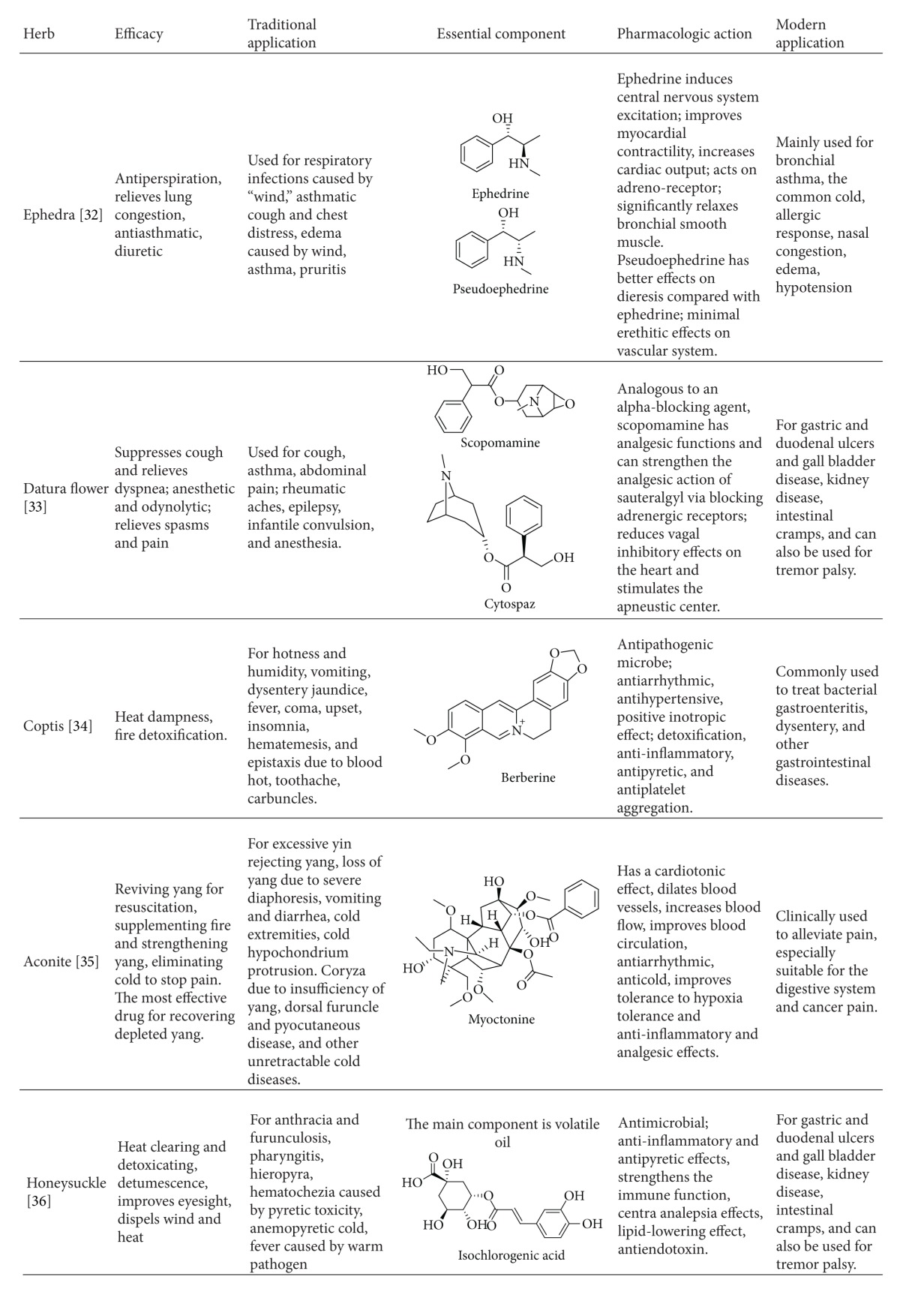

**Table 3:** Advances in traditional Chinese medicine that are ahead of modern medicine.

Traditional Chinese medicine (thousands of years ago)	Modern medicine (recent decades)
The integration of life knowledge/experience with traditional Chinese medicine	Ranging from primary studies of a single gene or protein to current use of genomics, proteomics and informatics

Emotional pathopoiesis and its correspondence between man and his environment	Gradual recognition of psychological factors and its relationship with physiology and pathology.

Syndrome differentiation, abidance by the triple pathogens and physique theory	Establishment of individual treatment

Chinese compound (multitarget herbal treatment)	From primary single target drugs to multitarget drugs, significantly reduce the side effects of medical treatment.

**Table 4:** Components and ingredients of the airway stabilizer solution.

Name	Herb	Ingredient
Airway stabilizer solution	Ephedra	Ephedrine and other minor components
	Ligusticum wallichii	Tetramethylpyrazine
	Ginkgo biloba	Ginkgolide
	Scutellaria	Baicalin
	Asarum	Racemic go A black alkali
	Ginger ale	Ginger oil glycosides
	Almonds	Amygdalin
	Earthworm	Earthworms antipyretic
	Rehmannia	Habitat catalpol
	Magnolia	Volatile oil

**Table 5:** Several advanced commonly used herbs.